# Preclinical Volume Retention of Fat Grafts Processed with REVOLVE™ Technology or Decantation Methods in Irradiated and Nonirradiated Wounds

**DOI:** 10.3390/jcm14093100

**Published:** 2025-04-30

**Authors:** Christopher A. Campbell, Graham M. Grogan, Samantha St. Jean, Nimesh Kabaria, Maryellen Gardocki-Sandor, Patrick S. Cottler

**Affiliations:** 1 Plastic Surgery, Maxillofacial & Oral Health, University of Virginia, Charlottesville, VA 22908, USA; cac5rb@uvahealth.org (C.A.C.); ggrogan@umc.edu (G.M.G.); 2 Center for Comparative Medicine, University of Virginia, Charlottesville, VA 22908, USA; dsq4ds@virginia.edu; 3 Allergan Aesthetics, an AbbVie Company, Branchburg, NJ 08876, USA; nimesh.kabaria@abbvie.com (N.K.); maryellen.sandor@abbvie.com (M.G.-S.); 4 Otolaryngology, Head and Neck Surgery, University of Virginia, Charlottesville, VA 22908, USA

**Keywords:** free fat transfer, adipose tissue processing, breast reconstruction

## Abstract

**Background:** The processing of harvested fat for transplantation is critical to fat graft performance. In breast reconstruction, larger volumes of fat are being grafted and, in some clinical cases, are being implanted within radiated tissue. This preclinical animal study evaluated the effects of radiation on retention volume and fat graft quality after processing by decantation or REVOLVE™ technology (Allergan Aesthetics, an AbbVie Company), a filtration-based device that can process lipoaspirates and remove unwanted contaminants prior to grafting. **Methods:** Lipoaspirate was collected from human donors (*n* = 6), processed using either REVOLVE™ technology or decantation, and implanted (0.5 cc) into 60 athymic mice for 4 weeks with or without a single 35-Gy radiation dose 12 weeks prior. Volume composition, MRI, and weight-based volumetric assessment of grafted fat were performed and compared between radiated and non-radiated mice. **Results:** Volume composition analysis demonstrated significantly higher fat content and lower aqueous fluid with REVOLVE™ technology than with decantation, with minimal cellular debris and free oil. MRI-based and weight-based volume analysis demonstrated a significantly higher percent retention with REVOLVE™ technology than decantation in nonirradiated and irradiated sites, respectively. Pathology scoring showed a significant decrease in fibrosis within grafts processed with REVOLVE™ technology in nonirradiated sites. **Conclusions:** Results suggest that fat processed using REVOLVE™ technology provides better early volume retention and quality of fat grafts compared to decantation, both in healthy and radiation-treated surgical sites.

## 1. Introduction

Fat grafting is a useful adjunctive procedure to breast reconstruction that effectively improves contour differences and soft tissue volume deficits, as well as increases soft tissue camouflage around breast implants to hide visible rippling and minimize implant palpability. Larger-volume fat grafting is being performed for breast augmentation and breast reconstruction, requiring larger-volume processing systems [1,2]. 

Post-operative fat graft volume loss has been reported to be up to 50% of the original grafted tissue in non-radiated breasts [3,4]. Clinical data suggest that the addition of the autologous stromal vascular fraction correlates with improved long-term fat graft volume retention [5]. In vivo studies have also corroborated the greater perceived clinical volume loss of grafted fat in patients that have required whole breast radiation as part of breast cancer treatment than in non-radiated surgical sites [6,7].

The performance of the REVOLVE™ platform technology in reducing contaminants while maintaining fat graft viability in an in vitro model has been studied [8]. Fat grafts harvested with the REVOLVE™ technology demonstrated higher populations of viable and functional adipocytes and stromal mesenchymal cells than decantation or centrifugation methods [9].

In vivo analysis of fat grafts prepared with REVOLVE™ technology [4] with and without radiation would provide an analysis of this processing technology in a model that is more clinically applicable to fat grafting for breast reconstruction and cosmetic indications [10,11].

To address this need, we evaluated fat graft composition after harvest with the REVOLVE™ technology, performed weight and MRI-based volume analysis in a small animal model with and without established radiation fibrosis following radiation therapy, and blinded pathologic analysis of explanted graft inflammation and adipocyte viability. This study design will help determine if fat graft volume retention is maintained following processing with REVOLVE™ technology in both radiated and non-radiated wound beds, as compared to the standard decantation method.

## 2. Materials and Methods

### 2.1. Human Adipose Tissue Harvest

All procedures were performed in accordance with the University of Virginia Institutional Review Board. At the time of large-volume elective liposuction, human adipose tissue was obtained from the abdomen and flank with the following technique [12,13,14,15]. Briefly, through a small incision, a mixed tumescent solution (0.5% lidocaine with 1:200,000 of epinephrine in Lactated Ringer’s solution) was infiltrated into the donor site using a blunt Lamis infiltrator (Byron Medical, Inc., Tucson, AZ, USA). The solution was infiltrated at a ratio of 1 cc of solution per cubic centimeter of fat graft to be harvested (tumescent technique). The adipose tissue was harvested through the same incisions made previously. Power-assisted liposuction (Microaire Surgical Instruments, Charlottesville, VA, USA) was performed with a flared 5 mm Mercedes cannula connected to a 2 L collection canister [10,11,12,13]. Upon completion, the canister that contained the lipoaspirate fat graft was transported to the University of Virginia Plastic Surgery Laboratory. Lipoaspirate (958 ± 469 mL) was obtained from 6 female patients (age range 32–61, BMI range 30.8–43.3).

### 2.2. Adipose Tissue Processing

#### 2.2.1. Decantation

Working in a sterile field, the lipoaspirate was thoroughly mixed within the canister to ensure that layering of lipoaspirate components had not occurred. Approximately 200 mL was transferred to a sterile jar, covered, and set at room temperature for 40 min to allow for decantation. The levels of the aqueous, adipose, and free lipid were marked on the jar, and the volume of each layer was recorded. The adipose layer was extracted and dispensed into 1 mL syringes in 0.5 mL aliquots, and the syringes were weighed.

#### 2.2.2. REVOLVE™ Technology Processing

For each donor, a separate sterile REVOLVE™ technology system was utilized. The system was connected via tubing to the appropriate destinations (lactated Ringer’s, vacuum, and harvest). Approximately 350 mL of lipoaspirate and an equal volume of lactated Ringer’s at 37 °C were transferred to the system, and the handle was rotated for 30 s. The waste was drained under vacuum (18 mmHg). The wash and drain steps were repeated until the waste returned clear. The enriched adipose tissue was removed from the system through the extraction port and dispensed into 1 mL syringes in 0.5 mL aliquots, and the syringes were weighed.

#### 2.2.3. Quantification of Graft Components Through Centrifugation

To characterize the composition of the grafts, 10 mL of tissue sample isolated from each of the processing techniques was placed into each of three 15 mL conical tubes. Samples were centrifuged at 300× *g* for 10 min to separate into the individual components. The volumes of the cell debris pellet, aqueous fluid, enriched adipose tissue, and free oil were recorded.

### 2.3. Preparation of Adipose Grafts

#### 2.3.1. Animal Model of Adipose Grafting

Animal experiments were performed under a protocol approved by the University of Virginia Institutional Animal Care and Use Committee (Animal Welfare Assurance A3245-01) in accordance with the National Institutes of Health’s Guide for the Care and Use of Laboratory Animals. Sixty female immunodeficient BALB/c Nude mice (19.8 ± 1.5 g) (Charles River, Raleigh, NC, USA, Strain 194) were used in this study (n = 10 for each clinical adipose tissue donor).

#### 2.3.2. Dermal Radiation

Thirty female immunodeficient BALB/c nude mice were exposed to a single dose of ionizing radiation across a dorsal skin flap as described previously [16]. The dorsal skin was extended and held away from the body, with a SARRP225 small-animal irradiator (Gulmay Medical, Inc., Suwanee, GA, USA). A 3 mm-thick lead shield was placed over the mouse so that only the skin flap was exposed. A 220-kV photon beam was used at 13.1 mA for 12.2 min, with the dose administered at 35 Gy. Mice were housed in an AAALAC-accredited facility. Within the dermis, irradiation induces inflammation, fibrosis, and vascular dropout that stabilizes 12 weeks post-radiation [17]; accordingly, biomaterial implantation was performed 12 weeks after irradiation, simulating delayed reconstruction [18]. During the 12-week post-radiation period, animals exhibiting ulceration were treated topically with Plurogel® burn dressing (Medline Industries, Northfield, IL, USA).

#### 2.3.3. Surgical Implantation of Adipose Grafts

Ten mice (n = 5, 0 Gy, 3 REVOLVE™ technology and 2 Decantation; n = 5, 35 Gy, 3 REVOLVE™ technology and 2 Decantation) received bilateral implants for each of the 6 adipose donors (Table 1). On the day of material implantation, animals were anesthetized with 2.0% inhaled isoflurane, and their dorsum (the site of radiation or analogous location on non-radiated animals) was prepped for aseptic surgery through the sequential application of chlorohexidine solution and 70% ethanol. Bilateral surgical incisions of 2 mm were created through the skin, laterally and perpendicular to the spine. A 1” 16-gauge angio-catheter was used to create subcutaneous pockets, just below the dermis, caudally to the incision. Next, 0.5 mL of adipose tissue was injected into each of the subcutaneous pockets. Incisions were closed with Dermabond. Empty syringes were weighed and compared to the loaded weights to calculate the precise graft volume. Animals received a subcutaneous injection of Ethiqa XR (buprenorphine-SR) 3.25 mg/kg (0.05 mL/20 mg) for pain relief following the implant surgery.

### 2.4. MRI Imaging

Animals receiving adipose grafts were imaged using contrast-free MRI at the time of implantation and a second MRI image at 4 weeks post-implantation to assess the graft volume. Scans were taken in a Bruker 9.4T BioSpec MRI system (Billerica, MA, USA) using the Dixon technique to identify the adipose tissue grafts [19]. The Dixon technique creates MRI images that are gated specifically to water and fat to render higher fidelity images and allow better evaluation of fat volume.

### 2.5. Volumetric Analysis of MRI Scans 

Volumetric rendering of the MRI images of the adipose grafts was performed using MicroDicom v.2022.2 (Sofia, Bulgaria). Adipose tissue was outlined on each individual DICOM image and integrated into a total volume.

### 2.6. Harvest of Adipose Tissue Grafts

At the terminal time point of four weeks post-implantation, mice were euthanized via anesthetic overdose. The skin above each graft and the adipose tissue were sharply dissected and carefully excised en bloc. An image of the adipose was taken to provide a macroscopic view of the graft. After imaging, one of the adipose grafts was carefully excised from the skin, and the weight was recorded. The bilateral graft was placed in 10% formalin for 48 h.

### 2.7. Histology and Immunohistochemistry

After fixation, adipose grafts were processed for paraffin embedding, and 5 μm samples were cut and stained with hematoxylin and eosin (H&E) and Masson’s Trichrome. Additional sections were immunohistochemically stained for CD31 for the presence of blood vessels. An established and widely applied scoring system was used to assess histologic parameters as follows: presence of intact and nucleated fat cells; presence of cysts and vacuoles; inflammation, as evidenced by infiltration of lymphocytes and macrophages; the presence of fibrosis and other components of connective tissue; and the level of adipocyte necrosis [20,21,22,23,24,25]. Each of these parameters was graded by a blinded veterinary pathologist on a scale of 0 to 4 by evaluation of the presence of the histologic parameters as follows: 0 = absence, 1 = minimal presence, 2 = mild presence, 3 = moderate presence, and 4 = marked/severe presence [26].

### 2.8. Statistical Analysis

All statistical analysis was performed in GraphPad Prism 10.2.3. Statistical significance was fixed below a cutoff of *p* < 0.05. Values for various metrics were compared between adipose tissue processed with the REVOLVE™ technology or decantation as well as between healthy and irradiated mice. Adipose graft composition, volume retention, weight retention, and each pathological score were evaluated using a two-way ANOVA and Bonferroni multiple comparison corrections.

## 3. Results

### 3.1. Adipose Tissue Processing

**Figure 1:**
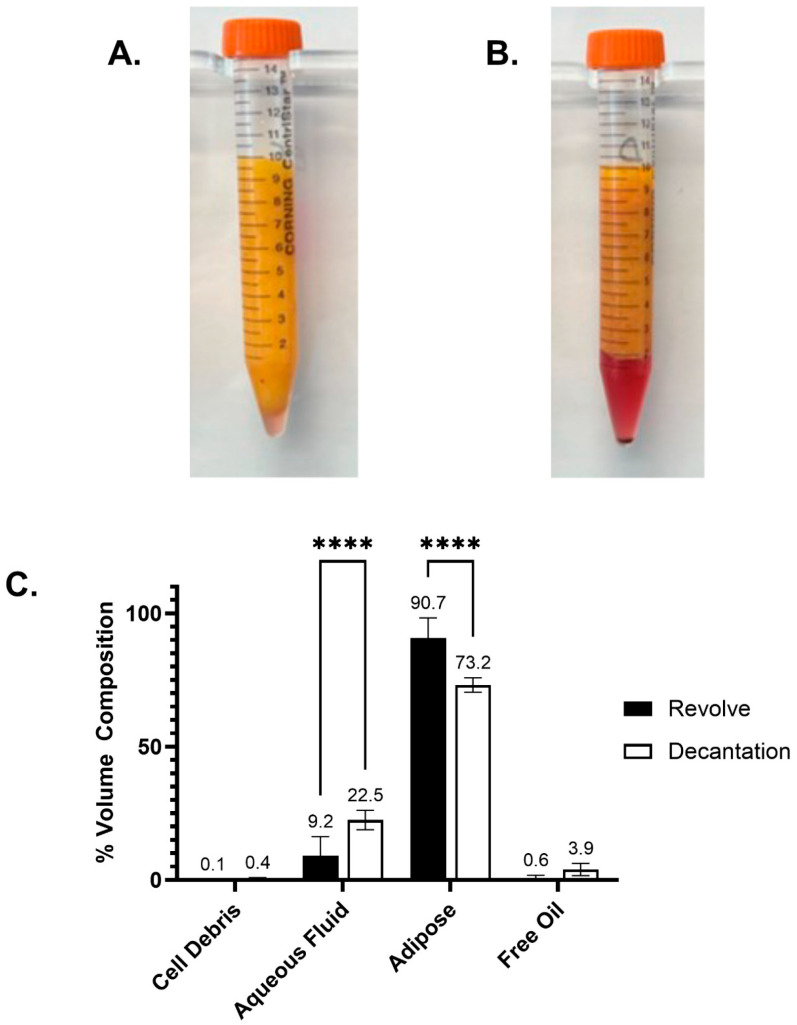
Adipose graft composition. Following processing of the lipoaspirate, the composition of the grafts (cellular debris, aqueous fluid, adipose tissue, and free lipids) was evaluated through centrifugation: (**A**) REVOLVE™ technology; (**B**) gravitational decantation. (**C**) REVOLVE™ technology resulted in significantly increased amounts of enriched adipose tissue and a significant decrease in the amount of aqueous fluid compared to decantation. Volume composition was evaluated through a two-way ANOVA followed by Bonferroni multiple comparison corrections (**** *p* < 0.0001).

Following centrifugation of the gravitationally decanted lipoaspirate yielded 0.4% cellular debris, 22.5% aqueous solution, 73.2% enriched adipose tissue, and 3.9% free oil. The REVOLVE™ technology processing of the adipose tissue resulted in 0.1% cellular debris, 9.2% aqueous solution, 90.7% enriched adipose tissue, and 0.6% free oil. The percentage of the aqueous solution was significantly lower (*p* < 0.0001), and the adipose tissue composition was significantly greater when processed by the REVOLVE™ technology (*p* < 0.0001) (Figure 1).

### 3.2. Preparation of Adipose Grafts

#### Surgical Implantation of Adipose Grafts

At the time of implantation, there was not a significant difference in the body weights of mice 12 weeks post-radiation compared to age-matched controls. All animals survived the implantation procedure and subsequent analysis.

### 3.3. Long-Term Graft Characteristics

**Figure 2:**
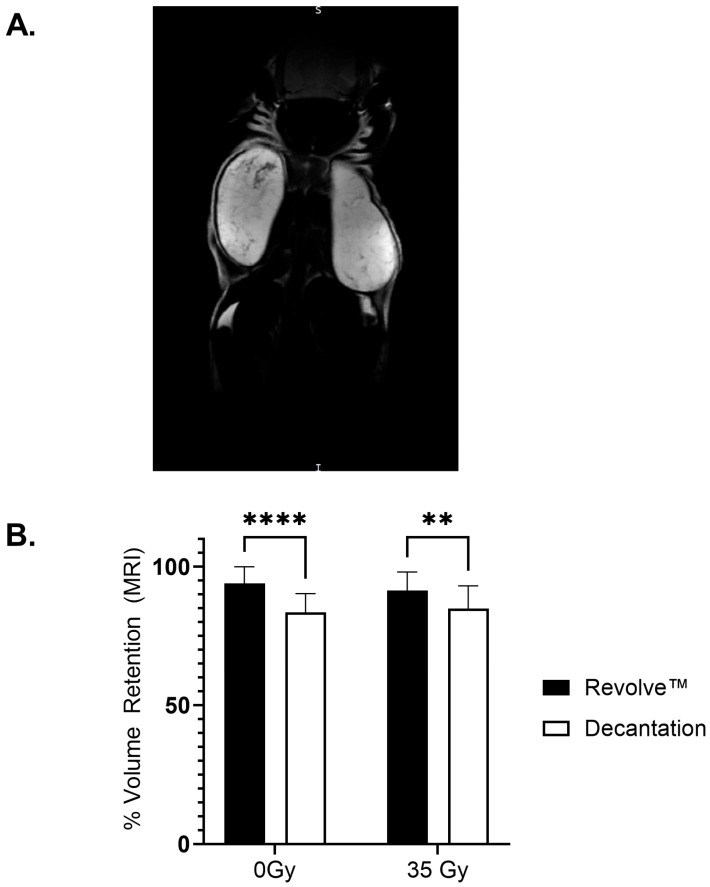
Volume retention. Non-destructive volume measurements of the adipose grafts were made at 4 weeks using MRI with the Dixon technique. (**A**) Representative MRI image showing the bilateral implants. (**B**) Percent volume retention was calculated for each group through comparison of the final to the initial graft values. Volume composition was evaluated through a two-way ANOVA followed by Bonferroni multiple comparison corrections. Grafts processed with REVOLVE™ technology demonstrated greater volume retention at 4 weeks than grafts processed by decantation (** *p* < 0.01; **** *p* < 0.0001).

Using the Dixon technique, the adipose grafts were delineated to ensure accurate volume rendering (Figure 2A). MRI analysis of the grafts at 4 weeks post-implantation showed that adipose tissue processed with the REVOLVE™ technology maintained a statistically significant higher in the percent retention of implant volume as compared to the decantation-processed adipose in both the non-radiated (93.9% ± 6.0% vs. 83.5% ± 6.7%; *p* < 0.0001, 0 Gy) and in the radiated groups (91.4% ± 6.7% vs. 84.8% ± 8.3%; *p* = 0.0004, 35 Gy). The effects of 35 Gy of radiation did not lead to significant volume differences in either the REVOLVE™ technology or decantation groups (Figure 2B).

**Figure 3:**
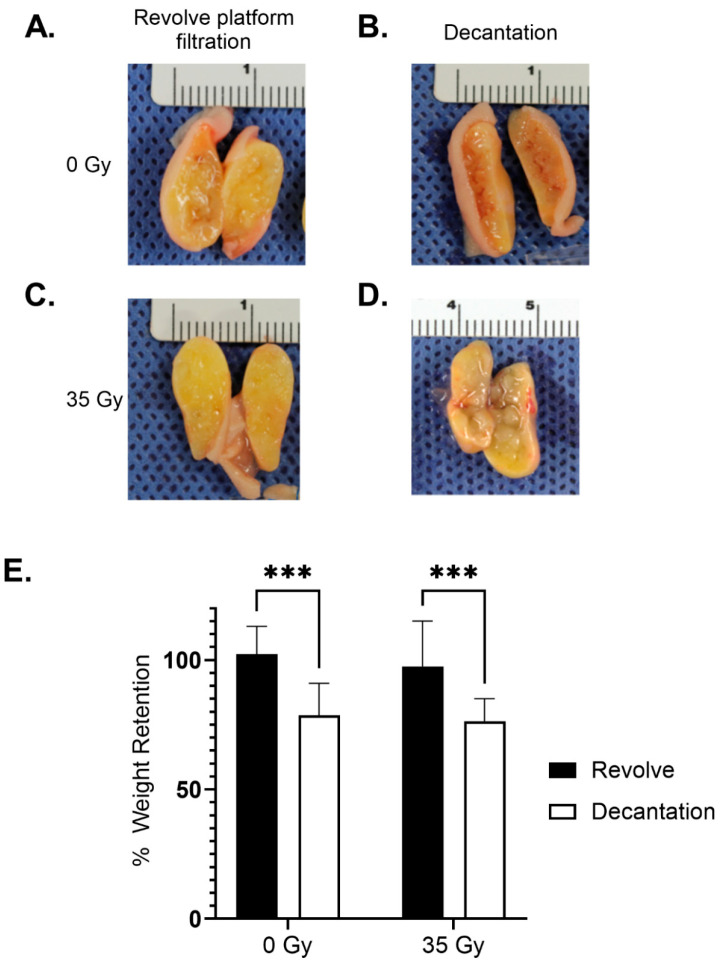
Weight retention. Carefully dissected grafts were grossly observed and weighed at 4 weeks and compared to the initial weight. (**A**–**D**) Representative images of grafts from the Revolve and Decantation processing at 0 Gy and 35 Gy. Harvested 4-week grafts processed with REVOLVE™ technology appear more uniform throughout with or without radiation than grafts processed with decantation. (**E**) Retention of initially grafted weight at the 4-week time point. Percent weight retention was evaluated through a two-way ANOVA followed by Bonferroni multiple comparison corrections, showing larger 4-week graft volume retained with prior REVOLVE™ technology processing (*** *p* < 0.001).

Four weeks post-implantation, the harvested grafts were visually inspected, weighed, and bisected. The REVOLVE™ technology-processed grafts exhibited a more uniform consistency and color compared to the grafts processed through decantation. In addition, adipose processed with decantation tended to be associated with more “cobblestone” consistency and free lipids, giving harvested grafts an oily appearance (Figure 3A–D). This factor could be associated with improved mechanical properties.

The weights of the grafts harvested at 4 weeks post-implantation, compared to the weight on the day of implantation, were consistent with the MRI volume data. At harvest, the REVOLVE™ technology-processed adipose grafts had weights that were statistically significantly higher as compared to the decantation-processed adipose in both the non-radiated (102.3% ± 10.7% vs. 78.6% ± 12.4%; *p* = 0.0001, 0 Gy) and in the radiated groups (97.6% ± 17.6% vs. 76.4% ± 8.7%; *p* = 0.0003, 35 Gy). The radiation level did not lead to significant volume differences in either the REVOLVE™ technology or decantation groups (Figure 3E).

### 3.4. Histology and Immunohistochemistry

**Figure 4:**
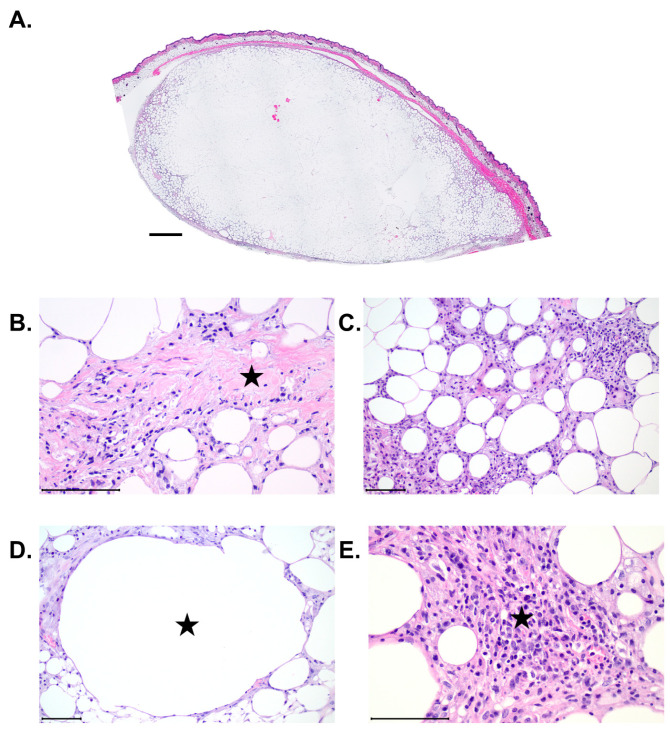
Pathology Examination. H&E-stained adipose grafts were evaluated for various characteristics of tissue health. (**A**) Representative cross section of adipose graft. The pale central area is attributed to autolysis due to histologic tissue processing. Scale bar = 1 mm. (**B**) Example image of Masson’s trichrome stain depicting the presence of fibrosis. 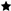 denotes area of fibrosis (blue collagen fibers). (**C**) Example of an H&E stain depicting the presence of adipocyte necrosis. 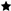 denotes necrotic adipocyte. (**D**) Example image of H&E stain depicting the presence of cysts and vacuoles. 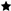 denotes cyst or vacuole. (**E**) Example image of H&E stain depicting the presence of inflammation. Star denotes presence of inflammation. Scale bar = 100 µm.

Each graft was sectioned and stained with hematoxylin and eosin (H&E) and imaged as an entire cross-section (Figure 4A). Higher magnification analysis was performed to evaluate the details of graft health (Figure 4B–E). There was evidence of minimal to moderate fibrosis, adipocyte necrosis, oil vacuoles, and inflammation seen in histologic analysis of H&E-stained slides (Figure 4B–E).

#### 3.4.1. Fibrosis 

**Figure 5:**
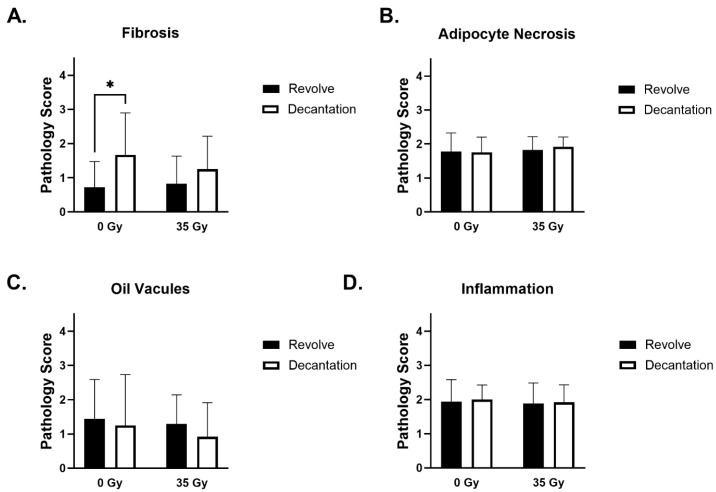
Pathology Scores. The grafts were evaluated for (**A**) fibrosis, (**B**) adipocyte necrosis, (**C**) oil vacuoles, and (**D**) inflammation. Each parameter was evaluated on H&E-stained slides and given a score of either Absent (0), Minimal (1), Mild (2), Moderate (3), or Marked/Severe (4) by a blinded pathologist. Average histological scores for each of the four metrics were analyzed for statistical differences; * = *p*-value < 0.05. Neither processing technique nor radiation exposure impacted the number of oil vacuoles (minimal), inflammation (moderate), or adipocyte necrosis (minimal to moderate).

Every graft evaluated contained a thin band of fibrous connective tissue surrounding it. Fibrosis was noted both peripherally and centrally and ranged from absent (0) to marked (4), with mean values between minimal and moderate. Within many of the grafts, there are distinct islands of connective tissue that are most likely intact connective tissue from the human adipose graft donors. These islands made it somewhat difficult to determine true fibrosis from donor connective tissue, as both are highlighted with Masson’s trichrome. Increased amounts of fibrosis are associated with increased inflammation and vascularization. Analysis of the pathology scoring revealed that the level of fibrosis with grafts processed with the REVOLVE™ technology was significantly reduced in non-irradiated mice compared to decantation processing (Figure 5A).

#### 3.4.2. Adipocyte Necrosis 

Individual to clumped necrotic adipocytes were seen mixed within the inflammatory cells on the periphery of the graft, but these areas are minimal (1) to mild (2) in severity and not the main histologic feature. There were not any significant differences in necrosis seen between the two processing techniques in either healthy or irradiated animals (Figure 5B).

#### 3.4.3. Oil Vacuoles

Oil vacuole formation ranged from absent (0) to marked (4), with mean values just greater than the mild designation. Oil vacuoles were most frequently found on the periphery of the graft, with larger vacuoles in the more marked cases appearing in the center. The severity of oil vacuoles appeared to increase with increased inflammation and fibrosis within the graft. There were no significant differences in the presence of oil vacuoles between the two processing techniques in either healthy or irradiated animals (Figure 5C).

#### 3.4.4. Inflammation

Inflammation was most often confined to the connective tissues surrounding the graft. The inflammatory infiltrate was composed mostly of mononuclear cells such as macrophages, lymphocytes, and plasma cells. Some of the animals also had an influx of neutrophils. Inflammation ranged from minimal (1) to moderate (3). More severe inflammation is associated with increased fibrosis and vascularization. There were no significant differences in inflammation seen between the two processing techniques in either healthy or irradiated animals (Figure 5D). 

#### 3.4.5. Adipocyte Size and Shape

Every graft evaluated showed heterogeneous sizes and shapes of adipocytes. Within each graft, the adipocytes in the center of the tissue are pale with nuclear fading/loss. No inflammatory cells or cellular debris are observed in these regions. Based on these histologic features, these areas have been interpreted as autolysis, which initiates immediately upon harvesting and is heavily dependent on the speed of tissue fixation, which may have played a contributing factor in this study (Figure 4A).

#### 3.4.6. Vascularization (CD31)

**Figure 6:**
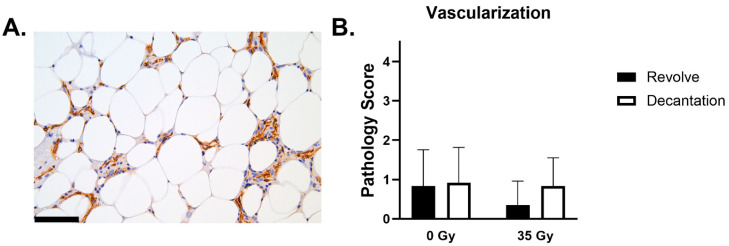
Graft vascularization. (**A**) Representative image of CD-31 staining demonstrating vascular investment into the adipose graft (brown = +CD31) (scale bar = 100 µm). (**B**) Average scoring of vascular presence within each graft: absent (0), minimal (1), mild (2), moderate (3), or marked (4).

Graft vascularization ranged from minimal to moderate, which is appropriate for adipose tissue given expected vascular density (3). All grafts had vessels around the periphery of the graft and sprinkled throughout (Figure 6A). In the animals with an increase in vascular profiles, these profiles were located either more peripherally, centrally, or both. This increase in vascular profiles is associated with areas of more severe inflammation and fibrosis. There were no significant differences in vascularization seen between the two processing techniques in either healthy or irradiated animals (Figure 6B).

## 4. Discussion

Fat graft harvest and grafting is a useful technique to improve patient satisfaction after breast reconstruction and is being used with increasing frequency in breast augmentation surgery [1,2,27]. Fat graft processing techniques and wound bed clinical characteristics can impact fat graft volume retention. Prior research has demonstrated that the REVOLVE™ technology reduces contaminants while maintaining graft viability in vitro [8]. Prior studies often do not include radiation therapy. The wound beds in these patients may be compromised due to radiation fibrosis [8,9]. 

In this study we demonstrated that harvested fat processed by the REVOLVE™ technology had a higher proportion of adipose tissue and fewer aqueous fluid or free oil contaminants than decanted specimens. This is consistent with prior work demonstrating that filtration systems yield a higher proportion of adipose tissue within harvested graft materials when compared to decantation or centrifugation [9]. 

A key aspect of this study was the inclusion of radiation to measure the effects of the resulting fibrosis on the outcomes of the fat grafts. The fat grafts were implanted into immunodeficient mice, and MRI images demonstrated increased volume retention in the REVOLVE™ technology groups over decantation in both irradiated and non-irradiated wound beds. Fat graft volume from imaging analysis correlated well with the weight of explanted specimens, corroborating MRI findings that specimens processed with REVOLVE™ technology had increased volume retention over decantation.

Fat grafting is often used for volume enhancement and aesthetic improvement after breast implant placement in radiated and non-radiated settings and to correct contour differences after breast conservation surgical therapy with radiation. Patients have significant variability in volume of fat graft retention based on differing wound bed characteristics, including radiation [6].

Adipose-derived stem cells harvested from mice after whole-body radiation demonstrated decreased cell number and decreased proliferation [6]. With concerns that grafted fat would not perform as well in radiated fields, we included a radiated group within our in vivo analysis. In our analysis, fat grafts placed within radiated and non-radiated animals had equivalent volume retention by MRI and post-explant weight. While murine models are not directly translatable to human physiology, the improved volume retention of fat grafts processed by filtration in the face of pre-existing radiation suggests that fat grafting may have clinical merit in improving outcomes in radiated surgical fields. 

Graft fibrosis and inflammation, cell size, shape, and necrosis were seen at rates consistent with prior analyses of fat grafts in murine models and were averaged at a mild (2) to moderate (3) rating on a scale of 0–4. The only significant difference between groups was the decreased fibrosis in non-radiated specimens processed with REVOLVE™ technology compared to decantation. In radiated fields, grafts processed by REVOLVE™ technology and decantation exhibited similar fibrosis levels between preparation groups. Moderate vascular levels were seen within the viable grafts and were similar for each between each processing method and radiation exposure.

Larger fat graft volumes are being utilized by plastic surgeons for clinical indications, including breast augmentation [28]. Fat processing techniques may impact clinical outcomes. In this study, we utilized contemporary clinical techniques to harvest high-volume fat for our grafts, including power-assisted lipectomy to increase clinical relevance. Specifically, the tumescent technique was used, as it has been shown to help mitigate donor site bruising and discomfort while not impacting fat graft viability [29]. The choice of anesthetic agent for tumescent technique (bupivacaine, ropivacaine, and lidocaine) has been shown to not detrimentally affect fat graft survival, so we maintained standard anesthetic usage [30]. Both abdominal and thigh donor sites were selected for harvest, as donor site has not been shown to impact retained fat graft volume [31]. When tumescent is used, there are no measurable differences in fat graft viability between different methods of fat graft harvest [32]. Decantation was chosen as a more appropriate comparison group, as centrifugation techniques can substantially alter cell structure and viability [33]. 

## 5. Conclusions

In this study, we used contemporary fat graft harvest techniques and compared the REVOLVE™ technology to decantation and found increased fat graft retained volume by MRI and explanted weight, likely due to the ratio and quality of graftable fat. Fat graft volume was preserved even when the animal was exposed to prior radiation. Future work will be needed to explore any potential benefits of fat grafting into radiated fields. In addition, histopathologic analysis showed grafts had lower fibrosis in the non-radiated field when the adipose was processed with the REVOLVE™ technology. These results show the potential of using the REVOLVE™ technology for the controlled washing of large-volume lipoaspirate as a reliable processing method for fat grafts used in clinical medicine. Study limitations include the difficulties extrapolating murine data to human clinical medicine and the need for additional longitudinal fat graft analysis at later time points.

## Figures and Tables

**Table 1:** Subject demographics and implant characteristics by treatment group.

	Revolve (0 Gy) *n* = 18	Revolve (35 Gy) *n* = 18	Decantation (0 Gy) *n* = 12	Decantation (35 Gy) *n* = 12	*p*
Mouse weight (g)	20.1 ± 1.2	19.3 ± 1.5	20.2 ± 1.5	19.6 ± 1.7	0.05
Implant weight (g)	0.59 ± 0.16	0.61 ± 0.07	0.56 ± 0.19	0.61 ± 0.06	0.05

## Data Availability

Data reported in this manuscript are available within the article. Additional data may be requested by contacting Allergan Aesthetics, an AbbVie Company, Branchburg, NJ, USA.
